# A systematic scoping review of the habitual dietary costs in low socioeconomic groups compared to high socioeconomic groups in Australia

**DOI:** 10.1186/s12937-020-00654-5

**Published:** 2020-12-10

**Authors:** Meron Lewis, Sarah A. McNaughton, Lucie Rychetnik, Amanda J. Lee

**Affiliations:** 1 School of Public Health, Faculty of Medicine, The University of Queensland, Herston, Queensland Australia; 2 The Australian Prevention Partnership Centre, The Sax Institute, Ultimo, New South Wales Australia; 3 Deakin University, Institute for Physical Activity and Nutrition, School of Exercise and Nutrition Sciences, Geelong, Australia; 4 School of Public Health, University of Sydney, Camperdown, New South Wales Australia

**Keywords:** Dietary intake, Low socioeconomic, Low income, Australia

## Abstract

**Background:**

Low socioeconomic groups (SEGs) in Australia are less likely to consume diets consistent with the Australian Dietary Guidelines (ADGs) and suffer poorer health than the broader population. The unaffordability, or perceived high cost, of healthy diets may be a factor. Detailed data on the cost of habitually consumed diets is required in order to inform strategies to alleviate socioeconomic impacts on dietary intake.

This systematic scoping review aims to identify the cost of the habitual dietary intake of low SEGs in Australia, in terms of the whole diet and its composite foods, in comparison to the cost in higher SEGs.

**Methods:**

A systematic search of peer-reviewed literature since 2000 and key government and non-government organisation (NGO) websites was undertaken. Data were extracted, synthesised and analysed in relation to study populations, dietary cost assessment measures, socioeconomic measures, and dietary cost and affordability.

**Results:**

The review identified four studies meeting inclusion criteria. Results confirmed that overall, low SEGs spend a lower amount, yet a higher proportion of household income, on food and drinks than higher SEGs. Quantitative comparison of the dietary costs between included studies was not possible due to difference in populations and study metrics. Costs of the habitual diet in these studies were not reported for ADG food groups, so did not allow for assessment of the healthfulness of the dietary intake or comparison with costs of recommended diets at food group level.

**Conclusions:**

Existing research does not provide sufficiently granular data of the costs of habitual diets of low SEGs in comparison to higher SEGs or data in a form that can inform strategies and interventions to improve dietary intake and diet-related health of low SEGs in Australia. Future empirical health research requires more granular measures of habitual spending on ADG food groups across SEGs.

## Background

Low socioeconomic groups (SEGs) are known to suffer poorer health than other population groups, with an estimated 2.1 year reduction in life expectancy in high income countries, including Australia, attributable to low socioeconomic status [1]. Food insecurity is also more prevalent in low SEGs [2], where people do not “at all times, have physical, social and economic access to sufficient, safe and nutritious food which meets their dietary needs and food preferences for an active and healthy life” [3].

In Australia, calls have been made for public policies to address the impacts of socioeconomic disadvantage in order to improve health for all [4–7]. Contributing factors include environmental, economic and social determinants [8]. In order to assess health inequities and identify low SEGs, estimates of income, occupational skill level or unemployment status, available household assets (e.g. car and home ownership), educational qualifications, and/or the clustering of these factors in specific locations may be used to classify population groups with common social or financial characteristics [9].

The contribution to poor health from diet is also large; whilst less than 1% of Australians follow the recommended Australian Dietary Guidelines (ADGs) [10, 11], there have been suggestions that low SEGs are more likely to consume a less healthy diet than the broader Australian population [12–14]. Cost and affordability are aspects of food choice which are of particular relevance to those receiving low incomes [14–16]. The household food budget is considered to be more flexible compared to other fixed household expenses (such as rent and utility bills), such that a household receiving a low disposable income that experiences a sudden additional expense is likely to lower their food budget to compensate [2]. A number of studies have examined the cost of ‘healthy’ food and assessed its affordability for low SEGs [17, 18], finding that ‘healthy’ diets, according to various definitions, are difficult to afford for low income households.

One indicator of unaffordability is when food costs 30% or more of disposable income [19–21]. More recently, 25% of disposable income has been posited as the level whereafter ‘food stress’ occurs [22]. High levels of food stress impact the ‘economic access’ dimension of food security, whilst other determinants of low socioeconomic status, such as car ownership and place of residence, also affect the dimensions of physical access, availability, and food utilisation [3].

Both real and perceived unaffordability of healthy diets may play an important role in determining the diets of low SEGs [23]. Consideration of the cost and affordability of healthy diets should be made in comparison to the cost of habitually consumed diets (which are likely to be less ‘healthy’) [19]. Habitually consumed diets comprise the types and amounts of food usually eaten on a regular basis, and are the major determinant of diet-related health [24].

A study of the Consumer Price Index (CPI) in Australia showed that over a period of 18 years healthier foods increased in price more than unhealthy foods [25]. Other studies have examined price per energy unit, concluding that foods high in nutrients, yet with a low energy density, were more expensive than less nutritious, energy dense foods [13, 19]. However, all of these studies have examined selected foods rather than whole diets or overall dietary patterns. Additionally, the data analysis of these studies includes energy in both metrics (price per kJ and kJ per gram) suggesting that the inverse relationship between the metrics may be a spurious mathematical artefact [13, 26]. Statistical analysis of the price of 4430 foods in the USA has indeed shown there is no support for the argument that high energy dense foods are cheaper than low energy dense foods [27].

Given lower incomes, low SEGs are likely to have less income to spend in absolute terms, and spend a higher proportion of their total income on their habitual diet, than higher SEGs [28]. However, it is not clear whether any studies have assessed habitual food costs of low SEGs with sufficient granularity to allow comparison to healthy food costs. Therefore, the aim of this systematic literature review is to identify studies assessing the cost of the habitual diet, and its composite foods, in low and higher SEGs in Australia and to determine whether comparisons can be made of relative diet quality.

## Methods

### Search strategy

The search strategy was structured to identify studies providing an assessment of the cost of habitual dietary intake of low SEGs in Australia in comparison to higher SEGs. The research question was considered in PICOT (population, intervention, comparator, outcome and time) format and the PRISMA (Preferred Reporting Items for Systematic Reviews and Meta-analyses) [29] statement was used to guide review processes.

The search *population* included Australians of any age and gender categorised as belonging to a low SEG by any method.

The *intervention* was defined as measurement of the cost of the habitual dietary intake of individuals or households of the search population. The *comparator* was the cost of the habitual dietary intake of individuals or households categorised as belonging to a higher SEG than the search population. *Outcomes* were defined as costs of habitual whole diets or selected food groups.

The search *timeframe* was restricted to documents published in the 20 years from July 2000 to October 2019, as it was considered that earlier documents may lack relevance due to changes over time in the social and economic landscape and dietary patterns of Australians. In particular, July 2000 saw the introduction of a goods and services tax in Australia, which exempts basic healthy foods.

The peer-reviewed literature databases searched were: The Cochrane Library; PubMed; MEDLINE; EMBASE; CINAHL; Informit Health Collection; and Web of Science (Science Citation Index and Conference Proceedings Citation Index). The search terms used were (nutrition OR diet OR diets OR food OR foods OR drinks) AND (cost OR costs OR costed OR price* OR afford*) AND (low-income OR low income OR low socioeconomic) AND Australia. “Low income” has been used commonly as a proxy for low socioeconomic status in research relating to the cost and affordability of healthy diets, and thus this term was used in the search as an additional term with ‘low socioeconomic’ [18, 20, 30]. Food costs and socioeconomic status are influenced by many country-specific social and economic issues, so the location was restricted to Australia as overseas findings would potentially be less applicable to the Australian situation.

Websites were identified as those known to the authors to provide information on diet cost or to report on the health and/or socioeconomic determinants of health in Australia [17, 30]. The websites searched were: The Australian Prevention Partnership Centre/The Sax Institute; Australian Health Policy Collaboration; Public Health Association of Australia; National Health and Medical Research Council; National Preventive Health Agency; Commonwealth Health Department; Australian Institute of Health and Welfare; Australian Treasury; Australian Council of Social Services; West Australian Council of Social Services; Queensland Council of Social Services; Victorian Council of Social Services; NSW Council of Social Services; South Australian Council of Social Services; Northern Territory Council of Social Service; Tasmanian Council of Social Services; and the Grattan Institute. Search terms ((Diet OR nutrition) AND (socioeconomic OR income)) were systematically entered into each website-specific search engine. The first five page returns, or the first ten items listed (when sorted by relevance) from each search was scrutinised. The results were screened using the inclusion and exclusion criteria described below.

The databases of peer-reviewed literature, and key government and non-government organisation related websites, were searched together with hand-searching all included references for any missing relevant documents. ML conducted all stages of the search and data extraction process, and AJL cross checked 50% of abstracts and data extractions to control for any observer bias.

#### Inclusion criterion

- Studies which described the individual or household habitual costs of food and drinks, and;
- Studies that differentiated the costs of food and drinks by a socioeconomic measure, and;
- Studies where the household or individual was located in Australia.

#### Exclusion criteria

- Any study not including individuals or households located in Australia, or;
- Any study solely relating to the cost of ‘healthy’ diets or other pre-determined specific diet that was not a habitual diet, or;
- Any study which did not report dietary costs differentiated by a socioeconomic measure, or;
- Any study solely qualitatively assessed the influence or perception of food price/affordability on purchasing behaviours

The listed databases and websites were searched and resulting citations were downloaded into EndNote X8 [31]. Duplicates were removed, and the inclusion and exclusion criteria were applied systematically to remaining citations, based on title, then abstract, then full text.

### Data extraction

Data were extracted by the following fields; study authors and date; age group, gender, and location of study population; measure of habitual dietary intake costs; SEG categorisation measure used; and, estimated dietary intake costs and proportion of income spent on dietary intake of each SEG. Quality assessment tools were not utilised as the included studies were descriptive studies and it was important to capture all available assessments of habitual diet costs. In this way, the methodology was similar to a scoping review.

### Data synthesis and analysis

Following data extraction, the representativeness of the included study’s population was assessed. The methods to assess diet cost and categorise SEGs used in the included studies were noted. Finally, the estimated dietary intake costs and proportions of income spent of each SEG were assessed for agreement between the included studies.

## Results

**Fig. 1:**
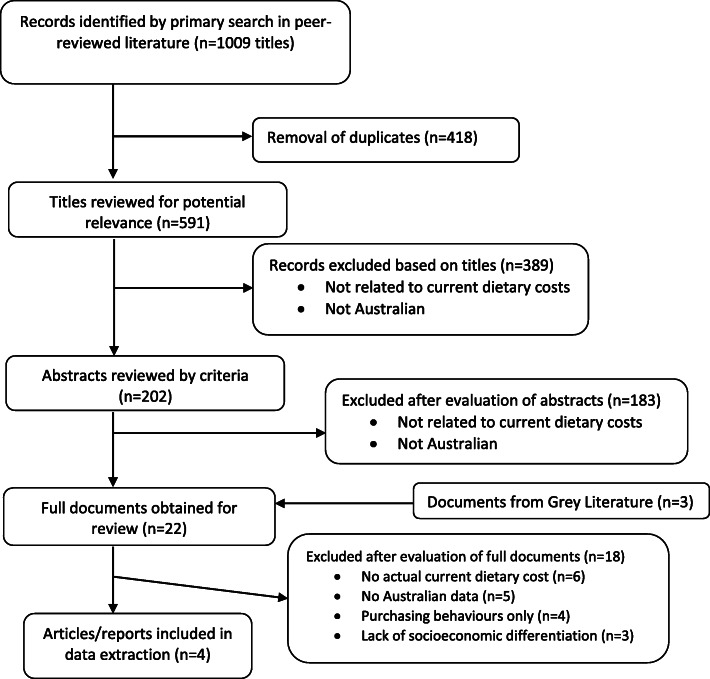
PRISMA Flowchart

Following application of the search strategy, one peer reviewed study (“Inglis et al.” [32]) and three large non-peer reviewed studies (Australian Bureau of Agriculture and Resource Economics and Sciences study “ABARES study” [33], Australian Bureau of Statistics Household Expenditure study “ABS HES study” [34], and The Household, Income and Labour Dynamics in Australia study “the HILDA study” [35]) were included. The PRISMA diagram is provided at Fig. 1. Data extraction from the included studies is provided at Table 1.

**Table 1:** Data extraction from included studies

Reference	Population studied Location & Date	Tool used to measure dietary costs	SEG categorisation tool used	Estimated mean weekly dietary costs of household reported ($)		Proportion of household income spent on dietary intake (%)	
				Lowest SEG (definition)	Highest SEG (definition)	Lowest SEG (definition)	Highest SEG (definition)
Inglis et al. [32]	Adult married women with 2 dependent children (*n* = 74) Melbourne, Australia, 2009	Typically purchased amounts of food & drink for a week selected from a list of 525 priced items	Household income^a^	$ 198.87 (Income <$1500/week)	$ 235.95 (Income >$1500/week)	n/a	n/a
ABARES study [33]	Households (*n* = 10,046) Australia-wide, 2015–16	Food & drink expenditure from 2 weeks of diary entries [36] Not included: food and drinks not further described, eggs and egg products, edible oils and fats or alcoholic beverages.	Gross household income	$ 114.15 (Lowest quintile)	$ 391.23 (Highest quintile)	n/a	n/a
			Household after tax income	n/a	n/a	24.8% (Lowest quintile)	10.5% (Highest quintile)
			Household net worth	$ 162.38 (Lowest quintile)	$ 324.19 (Highest quintile)	15.0% (Lowest quintile)	12.4% (Highest quintile)
			Main source of household income	$ 131.75 (Main income source is government pension or allowance)	$ 270.18 (Main income source is private income)	19.0% (Main income source is government pension or allowance)	13.0% (Main income source is private income)
ABS HES study [34]	Households (*n* = 10,046) Australia-wide 2015–16	Total food & drink expenditure from 2 weeks of diary entries [36]	Equivalent^b^ household disposable income	$ 156.66 (Lowest quintile)	$ 398.02 (Highest quintile)	n/a	n/a
			Household net worth	$ 182.05 (Lowest quintile)	$ 373.19 (Highest quintile)	n/a	n/a
			Main source of household income	$ 143.29 (Main income source is government pension or allowance)	$ 309.02 (Main income source is private income)	n/a	n/a
HILDA Study [35]	Approx. 9800 households Australia-wide 2001–2012	Total food & drink expenditure from self-filled questionnaire including: weekly groceries, alcohol & meals eaten out	Equivalent^b^ household disposable income	$ 187.56^c^ (Lowest quintile)	$ 283.28^c^ (Highest quintile)	26.6%^d^ (Lowest quintile)	9.2%^d^ (Highest quintile)

^a^Unknown if gross or disposable income

^b^Household disposable income was adjusted to account for different household sizes and age of household members (ABS) [37]

^c^Reported data from 2012 [35]

^d^Result aggregated from data collected 2001–2012 [38]

The ABARES study and the ABS HES study both provide analysis of the Household Expenditure Survey (HES) dataset produced by the Australian Bureau of Statistics [34]. The Household Expenditure Study is conducted approximately every 5 years, so only the most recently available data from 2015 to 16 were included.

### Population

The study by Inglis et al. was small (*n* = 74), and restricted to households including a married couple and two dependent children residing in the major city of Melbourne, Australia (Table 1) [32]. Data was reported for the household by the adult women of the family [32]. In contrast, the ABARES, ABS HES and HILDA studies were large (*n* ≈ 10,000) and analysed data from households Australia-wide [33–35].

### Tool used to measure habitual dietary costs

Inglis et al. used a tool where participants selected amounts typically purchased from a priced list of 525 foods and drinks (Table 1) [32]. Similarly, the HILDA study administered a retrospective questionnaire collecting participants’ retrospective recall of their habitual spending in the categories of: food and drinks; alcohol; and meals eaten out [39]. The HES dataset, analysed by the ABS HES and ABARES studies, was collected via a prospective diary method, where participants recorded all spending over a 2 week period, either manually or by providing store dockets [40].

Habitual dietary cost data were also reported as separate costs for various food categories by the ABS HES and ABARES studies [34]. However, the categorisation used was a historical, commodity/culinary-based system that does not align with the ADGs’ groupings [10]. For example: cakes and biscuits were included in the category of bakery products, flour and cereals; processed meat, bacon & sausages were included in the category of meats; flavoured milk, cream and butter were included in the category of dairy. All of these example foods are classified by the ADGs as discretionary foods (those not necessary for health that are high in saturated fat, salt, added sugar, and/or alcohol) [10]. Additionally, the categories reported in the ABARES study were different to those of the ABS HES, with some categories combined (e.g. fruit and vegetables) and others omitted (e.g. alcoholic drinks).

### SEG categorisation tool used

All four included studies categorised SEG by a financial variable (Table 1). Household income was used in all studies, either as gross income, after tax income, equivalent disposable income, or without further definition. Equivalent disposable household income was calculated by adjusting the reported actual household income to account for the number and age (greater than or less than 15 years) of household members [37].

Household net worth was also used to categorise SEGs in the ABS HES and ABARES studies [33, 34]. In these two studies, SEG was additionally categorised by the reported main source of household income. Private income, such as employment or investment income, was distinguished from government pensions or allowances, such as unemployment benefits, single parent pension, disability pension, and aged pensions [33, 34].

### Estimated dietary costs

All four studies reported quantified dietary costs by SEGs, and all found that lower SEGs had lower dietary costs than higher SEGs (Table 1) [32–35]. The estimated mean weekly dietary cost of the lowest SEGs varied from $114.15 [33] to $187.56 [35] per household. The highest SEGs’ estimated mean weekly dietary cost varied from $235.95 [32] to $398.02 [34] per household. Whilst the ABARES study [33] analysed the same data set as the ABS HES study [34], it did not report the categories of “food and drinks not further described”, “eggs and egg products”, “edible oils and fats” or “alcoholic beverages”, resulting in lower estimated dietary costs than expected. The Inglis study [32] only reported costs of a family of four (two adults and two children), whereas the other three studies [33–35] reported dietary costs equivalised from a range of household sizes.

### Proportion of household income spent on dietary costs

The ABARES and HILDA studies reported the proportion of household income spent on food and drinks (Table 1) [33, 35]. The HILDA study reported that 34% of all households in the bottom two quintiles of household income distribution spent more than 30% of household income on food [35]. It is unclear if this proportion had been calculated on gross or disposable income.

The ABARES study reported that, using household after tax income as a socioeconomic measure, the lowest quintile spent 24.8% of income on dietary intake, whereas the highest quintile spent 10.5%. When this study used household net worth as a socioeconomic measure, the lowest quintile spent 15.0% of income on dietary intake, whereas the highest quintile spent 12.4%. Further, when this study reported dietary costs using the main source of household income as the socioeconomic measure, households mainly receiving government pensions or allowances spent 19.0% of income on dietary intake, whereas households mainly receiving income from private sources spent 13.0% on diet.

### Estimated dietary cost by food category

In the two included studies reporting expenditure by food categories (Table 2), the lowest SEG (by any measure) spent less in absolute costs than the highest SEG in almost all food categories [33, 34]. The exception was reported in the ABARES study, where expenditure on dairy products was slightly higher for the lowest quintile of gross household income than the highest quintile [33]. The main differences in spending patterns by SEG were for the categories of ‘meals out’ and fast foods [33, 34], where those households in the lowest SEG assessed by any measure spent a significantly lower proportion of the total food expenditure on these categories than higher SEGs. Similarly for the category of alcoholic drinks, where provided [34], the lowest SEGs spent a much lower proportion of total food expenditure on these than higher SEGs.

**Table 2:** Expenditure per food category by SEG as reported only in two of the four included studies

Food category	Weekly Expenditure per household ($) - ABS HES [34]						Annual Expenditure per person ($) - ABARES [33]					
	Equivalised Household Income		Household Net Worth		Household Main Source of Income		Gross Household Income		Household Net Worth		Household Main Source of Income	
	Lowest Quintile	Highest Quintile	Lowest Quintile	Highest Quintile	Government Pensions & Allowances	Private income	Lowest Quintile	Highest Quintile	Lowest Quintile	Highest Quintile	Government Pensions & Allowances	Private income
Food and non-alcoholic beverages not further described	$9.93	$20.77	$12.79	$18.11	$6.81	$17.92	n/a	n/a	n/a	n/a	n/a	n/a
Bakery products, flour and cereals	$14.59	$22.78	$13.42	$25.55	$14.18	$21.64	$417	$437	$310	$464	$377	$401
Meat (excluding fish and seafood)	$19.61	$32.26	$17.78	$35.46	$18.53	$29.71	$669	$722	$486	$808	$595	$662
Fish and seafood	$4.11	$7.52	$3.27	$9.04	$3.86	$5.96						
Eggs and egg products	$1.56	$2.31	$1.43	$2.29	$1.37	$2.01	n/a	n/a	n/a	n/a	n/a	n/a
Dairy products	$11.62	$17.58	$10.49	$19.14	$11.2	$16.34	$328	$317	$242	$347	$298	$303
Edible oils and fats	$1.29	$1.47	$1.08	$1.74	$1.45	$1.51	n/a	n/a	n/a	n/a	n/a	n/a
Fruit and nuts	$10.22	$19.42	$8.69	$22.7	$9.94	$16.28	$633	$674	$433	$790	$550	$613
Vegetables	$11.39	$19.28	$10.07	$20.82	$10.74	$16.76						
Condiments, confectionery, food additives and prepared meals	$18.20	$31.30	$18.99	$31.20	$18.06	$27.92	$513	$560	$438	$566	$480	$518
Non-alcoholic beverages	$10.36	$18.51	$11.69	$17.90	$9.79	$16.67	$287	$335	$270	$325	$260	$309
Meals out and fast foods	$31.64	$146.58	$51.91	$121.55	$25.54	$97.67	$913	$2406	$1198	$2207	$679	$1812
Other food and non-alcoholic beverages	$0.14	$0.35	$0.11	$0.22	$0.17	$0.24	$318	$451	$371	$378	$263	$394
Alcoholic beverages	$12.00	$57.89	$20.33	$47.47	$11.65	$38.39	n/a	n/a	n/a	n/a	n/a	n/a
**Total**	**$156.66**	**$398.02**	**$182.05**	**$373.19**	**$143.29**	**$309.02**	**$4078**	**$5902**	**$3748**	**$5885**	**$3502**	**$5012**

### Proportion of total food expenditure per food category

The ABARES study also reported the proportion of the total food expenditure for each food category, and similar data were calculated from the reported ABS HES expenditure data (Table 3). Again, the largest difference between SEGs by any measure was the category of ‘meals out’ and fast food and the category of alcohol, where reported; the higher SEGs spent a much greater proportion of their total food expenditure on these items than the lowest SEGs.

**Table 3:** Proportion of total food expenditure per food category by SEG from the two of the four included studies that reported this^a^

Food category	Proportion of total food expenditure per food category – ABS HES						Proportion of total food expenditure per food category - ABARES study [33]					
	Gross Household Income		Household Net Worth		Household Main Source of Income		Equivalised Household Income		Household Net Worth		Household Main Source of Income	
	Lowest Quintile	Highest Quintile	Lowest Quintile	Highest Quintile	Government Pensions & Allowances	Private income	Lowest Quintile	Highest Quintile	Lowest Quintile	Highest Quintile	Government Pensions & Allowances	Private income
Food and non-alcoholic beverages not further described	6.3%	5.2%	7.0%	4.9%	4.8%	5.8%	n/a	n/a	n/a	n/a	n/a	n/a
Bakery products, flour and cereals	9.3%	5.7%	7.4%	6.8%	9.9%	7.0%	10.2%	7.4%	8.3%	7.9%	10.8%	8.0%
Meat	12.5%	8.1%	9.8%	9.5%	12.9%	9.6%	16.4%	12.2%	13.0%	13.7%	17.0%	13.2%
Fish and seafood	2.6%	1.9%	1.8%	2.4%	2.7%	1.9%						
Eggs and egg products	1.0%	0.6%	0.8%	0.6%	1.0%	0.7%	n/a	n/a	n/a	n/a	n/a	n/a
Dairy products	7.4%	4.4%	5.8%	5.1%	7.8%	5.3%	8.0%	5.4%	6.5%	5.9%	8.5%	6.0%
Edible oils and fats	0.8%	0.4%	0.6%	0.5%	1.0%	0.5%	n/a	n/a	n/a	n/a	n/a	n/a
Fruit and nuts	6.5%	4.9%	4.8%	6.1%	6.9%	5.3%	15.5%	11.4%	11.6%	13.4%	15.7%	12.2%
Vegetables	7.3%	4.8%	5.5%	5.6%	7.5%	5.4%						
Condiments, confectionery, etc	11.6%	7.9%	10.4%	8.4%	12.6%	9.0%	12.6%	9.5%	11.7%	9.6%	13.7%	10.3%
Non-alcoholic beverages	6.6%	4.7%	6.4%	4.8%	6.8%	5.4%	7.0%	5.7%	7.2%	5.5%	7.4%	6.2%
Meals out and fast foods	20.2%	36.8%	28.5%	32.6%	17.8%	31.6%	22.4%	40.8%	32.0%	37.5%	19.4%	36.1%
Other food	0.1%	0.1%	0.1%	0.1%	0.1%	0.1%	7.8%	7.6%	9.9%	6.4%	7.5%	7.9%
Alcoholic beverages	7.7%	14.5%	11.2%	12.7%	8.1%	12.4%	n/a	n/a	n/a	n/a	n/a	n/a
Total	100%	100%	100%	100%	100%	100%	100%	100%	100%	100%	100%	100%

^a^ABS HES proportions calculated from reported data, ABARES proportions as reported

## Discussion

This systematic review examined studies assessing the cost of the habitual diet, and its composite foods, in low and higher SEGs in Australia. The review identified only four studies that met the inclusion criteria. All included studies found that low SEG households (categorised by various financial variables) spent less on their diet than higher SEG households [32–35]. However, a lack of reporting of costs by ADG food groups excluded potential quantitative analysis of habitual diet costs by healthy or unhealthy food and drinks categories.

The small number of identified studies from this systematic literature review was surprising, given the frequent identification in the literature of the importance of price on food choice [15, 23, 38, 41, 42]. Research on the cost of ‘healthy’ foods and whether these are affordable for low SEGs has been conducted in Australia, but few studies have examined or compared the costs of healthy diets to habitual total food and drink expenditure by these groups [17].

Previous analysis of household cost and expenditure surveys internationally, found a large degree of heterogeneity in the measurement methods and purpose of the surveys, and limitations in the application of such survey results to nutrition policy [43]. The most recent AHS NNPAS in 2011–13 [11] used food categories aligned with the food groups of the ADGs [10]. Similar re-categorisation within the food and drink expenditure section of the HES [34] would provide harmonious data that support more meaningful analysis of monitoring and surveillance data sets from a nutrition and health perspective. A concordance between the food categories of the HES (also used in calculation of Consumer Price Index (CPI) information) and the ADG food groups has been developed and used by the ABS [44], however it is not publicly available.

### Under-representation of rural and remote populations

Three of the four studies included in this review analysed data from a large national sample from throughout Australia [33–35]. Such Australia-wide studies are designed with the intention of being nationally representative, however they do not include participants from communities classified as ‘very remote’ [40]. Additionally, the small sample numbers of those living in rural and remote locations, makes examination of the impact of location on dietary cost challenging. Whilst location and remoteness were not a focus of this review, rural and remote locations tend to include a high proportion of low SEGs [9], and contend with higher food prices, and poorer availability and access, compared to major cities [17].

### Challenges of implementing habitual dietary costs measurement tools

Research into financial affairs of the population can be challenging due to a reluctance for participants to discuss private matters such as their family finances [45]. Social desirability bias may affect the accuracy of reported food purchasing, similarly to dietary intake reporting, where consumption of healthy foods is commonly over-reported and less healthy foods under-reported [46]. Data collection can be onerous for participants, especially when required to identify and record all spending. For example, the ABS HES requires survey participants to diarise all expenditure for 2 weeks [36]. The method used by Inglis et al., involving selection of frequency of purchase of costed products from a list of items, excludes non-supermarket items such as takeaway or restaurant foods, and alcohol, which may explain the lower expenditure estimates of this study compared to those reported in the ABS HES [32]. Further, this method does not capture the relatively lower spending of low SEGs in these categories compared to higher SEGs, and thus the differential in expenditure between SEGs is lower than in the other included studies. Additionally, for those households experiencing food insecurity, food acquired from sources such as family members or charities will not be captured by food expenditure records, as it does not involve a financial cost. Less onerous data collection techniques, such as the use of barcode scanning of food purchases [47], may improve accuracy of food cost records.

### Limitations of tools used to categorise SEG

Categorisation of population groups by socioeconomic status may utilise one or more of a variety of measures, each of which affect the findings. When household income is used to categorise SEGs, it should be noted that the lowest income quintile, as defined by the ABS HES and the HILDA datasets [34, 35], is likely to contain older person households who are retired with a low income, but have access to lifetime savings for daily expenditure. This is evidenced by the higher net worth of the lowest quintile than the next three higher income quintiles, and that the total goods and services expenditure of the lowest quintile is 137% of after tax income [33]. Similarly, when categorising SEGs by net worth, the lowest quintile will include households of younger people who may have “reasonable incomes but have yet to accumulate significant assets” [33]. Low household worth may also occur when assets are offset by high levels of debt [48]. High household net worth can provide reserves to support expenditure, but may also be bound in assets that limit cash flow [48]. In Australia, household income is more equally distributed than household wealth [49]. All of these limitations suggests that net worth is a blunt instrument for categorisation of SEGs for the purposes of investigating expenditure on diet, which relies upon accessible funds on a regular basis.

Those households mainly receiving income from government pensions and allowances include groups such as the unemployed, single parents, people with a disability, and retirees without significant private funds, all of whom could be considered as low income due to pension/allowance rates usually being lower than a workforce income. The categorisation of SEGs by income source is not ideal however, as income from private sources may include minimum wage, or under-employment, and thus result in a low income.

Inglis et al. used a household income level of less than or more than $1500/week to discriminate between high and low SEGs [32]. It was not reported if this was gross or disposable household income. This cut point appears consistent with the mean disposable income per household in the middle tertile of Australia in 2009 ($721/week equivalised household income = $1514 for a family of four) [50].

### Comparison of estimated dietary costs

Overall, the four included studies found that low SEGs (measured by various financial variables) spent less on their diet than higher SEGs [32–35]. The heterogeneity of the study populations, SEG categorisation and food expenditure measures preclude direct quantitative comparison between the included studies. A large difference was observed between the lowest and highest SEGs in spending on meals out and fast food both in actual cost [34] and proportion of total food expenditure [33]. However, this commodity/culinary based categorisation of foods does not necessarily indicate a difference in the healthfulness of food consumed, merely difference in the purchase location. None of the included studies reported food expenditure by food groups consistent with the ADGs groupings [10]. Whilst it is known from population dietary intake data that low SEGs consume lower quality diets than higher SEGs [12], the lack of specific data supporting assessment of the healthfulness of the costed diets means that it is currently unclear if low SEGs, or any population group, will need to spend more or less on various food groups and total diets in order to move towards a healthier diet.

### Proportion of income spent on diet

The included studies reported that the proportion of household income spent on food and drinks was, as expected, consistently higher for low SEGs [33, 35]. The ABARES report, using household after tax income to categorise SEG, found that expenditure on food and drinks by the lowest SEG was close to 25% of household income. Similarly, the HILDA study found that a significant proportion of households (34%) in not just the lowest, but the two lowest quintiles of household income, had expenditure above 30% of household income. Thus households of low SEGs are likely to have difficulty affording their habitual diet. Indeed, single-item measures of food insecurity in Australia [51] suggest 4% of households are affected, but more comprehensive measures encompassing all dimensions of food insecurity estimate prevalence at 10–30% of households [52].

Given this, the development of public health policy to encourage the purchase and consumption of healthier food and drinks must consider cost implications, as any actual or perceived need for additional food expenditure is likely to be rejected by those who cannot stretch their budget any further. In particular, research comparing the cost of healthy diets to habitual diet costs of low SEGs would provide strong evidence to support such policies.

### Strengths and limitations of the review

A strength of this review is detailed analysis of the factors reported in the included studies that influence assessment of the habitual dietary costs of Australians differentiated by SEGs. The review was limited by the availability of documents to online searches, and to the data reported. The HES and HILDA surveys were not designed primarily for dietary analysis and the current reporting formats do not support this in any detail.

A limitation of studies of habitual food expenditure is that cost differences due to both brand or food type choice are not assessed, and thus a higher cost of a food category does not necessarily reflect a higher intake. For example, the unit expenditure on fruit will be higher for out-of-season produce compared to the same quantity of in-season fruits, and usually unit expenditure on branded products will be higher than on home-label, generic items. A more recently developed alternative method uses reported intake data from dietary surveys to determine the mean intake of a population group, followed by costing of this diet using the most common brands or food types [30, 53]. This standardised approach, the Healthy Diets Australian Standardised Affordability and Pricing (ASAP) methods protocol, therefore provides a more comprehensive assessment of the relationship between diet cost and health [30].

Due to the small number and heterogeneous nature of the identified studies, meta-analysis or other statistical analysis of the results was precluded. Future research in this area that includes harmonized, granular data aligned with the ADG food groups would provide stronger evidence of the relationship between expenditure on dietary intake by SEG from a health perspective.

## Conclusions

This systematic review confirmed that there were differences in expenditure on dietary intake by SEG; all included studies reported that low SEGs spent less on diet than higher SEGs in absolute terms, but spent a higher proportion of their household income on food and drinks. Heterogeneity due to differences in populations studied, SEG categorisation and methods of measuring and reporting dietary intake costs did not allow quantitative nutritional analysis of dietary intake costs across the included studies.

A lack of granularity of data and lack of reporting of the cost by ADG food group expenditure meant that the results could not be used to assess habitual household expenditure on healthy or unhealthy foods and drinks. Updating the food categories used in national household expenditure surveys to align with the ADG food groups would assist in the provision of detailed data that can be synthesised to support the development of targeted interventions and policies to reduce the inequities of healthy eating.

## Data Availability

Data sharing is not applicable to this article as no datasets were generated or analysed during the current study.

## Abbreviations

ABARES: Australian Bureau of Agriculture and Resource Economics and Sciences
ABS HES: Australian Bureau of Statistics Household Expenditure Study
ADG: Australian Dietary Guidelines
CPI: Consumer Price Index
HILDA: Household, Income and Labour Dynamics in Australia
PRISMA: Preferred Reporting Items for Systematic Reviews and Meta-analyses
SEG: Socioeconomic group
